# Environmental interventions to support orientation and social engagement of people with Alzheimer’s disease

**DOI:** 10.1590/1980-57642021dn15-040012

**Published:** 2021

**Authors:** Maria Carolina Dias de Azevedo, Helenice Charchat-Fichman, Vera Maria Marsicano Damazio

**Affiliations:** 1 Arts & Design Department, Pontifícia Universidade Católica do Rio de Janeiro – Rio de Janeiro, RJ, Brazil.; 2 Psychology Department, Pontifícia Universidade Católica do Rio de Janeiro – Rio de Janeiro, RJ Brazil.

**Keywords:** Alzheimer’s disease, dementia, interior design and furnishings, evidence-based facility design, doença de Alzheimer, demência, decoração de interiores e mobiliário, projeto arquitetônico baseado em evidências.

## Abstract

The built environment can be a home to compensatory strategies aimed at increasing the independence of elderly people with Alzheimer’s disease, by mitigating the cognitive impairment caused by it.

**Objective:**

The aim of this study was to find out which interventions were performed in indoor environments and observe their impacts on the relief of behavioral symptoms related to the disorientation of elderly people with probable Alzheimer’s disease.

**Methods:**

A systematic review was carried out using the preferred reporting items for systematic review and meta-analyses criteria in the MEDLINE/PubMed database. Two researchers carried out the selection of the studies, following the same methodology. The third author contributed during the writing process and in the decision-making.

**Results:**

Of note, 375 studies were identified and 20 studies were included in this systematic review. The identified interventions were classified into environmental communications and environmental characteristics.

**Conclusions:**

Environmental communications had positive results in guiding and reducing agitation. In contrast, while reducing behavioral symptoms related to orientation, environmental characteristics showed improvements mainly in social engagement and functional capacity.

## RESUMO

O ambiente construído pode ser lar para estratégias compensatórias destinadas a aumentar a independência de idosos com doença de Alzheimer, ao mitigar o comprometimento cognitivo causado por ela.

**Objetivo:**

Descobrir quais intervenções foram realizadas em ambientes internos e observar seus impactos no alívio de sintomas comportamentais relacionados à desorientação de idosos com provável doença de Alzheimer.

**Métodos:**

Uma revisão sistemática foi realizada usando os critérios Preferred Reporting Items for Systematic Reviews and Meta-Analyses na base de dados Medical Literature Analysis and Retrieval System Online/ PubMed. Dois pesquisadores realizaram a seleção dos estudos seguindo a mesma metodologia. O terceiro autor contribuiu durante o processo de redação e nas tomadas de decisão.

**Resultados:**

Foram identificados 375 estudos e 20 foram incluídos na presente revisão sistemática. As intervenções observadas foram classificadas em comunicações ambientais e características ambientais.

**Conclusões:**

As comunicações ambientais tiveram resultados positivos na orientação e na redução da agitação. As características ambientais, por outro lado, embora reduzam os sintomas comportamentais relacionados à orientação, mostraram melhorias principalmente no engajamento social e na capacidade funcional.

## INTRODUCTION

Dementia is an umbrella term for several diseases which affects the brain in a way that compromises one’s cognitive processes, behavior, and ability to carry on daily tasks.^1^ It is claimed to be the “major cause of disability and dependency among older adults worldwide.”^2^ Under those circumstances, the number of people diagnosed is increasing: from the estimated 50 million in 2019, the scale of the issue becomes three times worse, rising to 152 million people in 2050.^1^ Alzheimer’s disease (AD) is the most common form of dementia, contributing to 60–70% of the total cases.

Although dementia is related to the progressive and global decline of cognition, there are some skills and abilities that can still be accessed through design.^3^ In this spectrum, the role that the built environment plays in supporting people with dementia can be either therapeutic or debilitating: it can be a home for compensatory strategies designed to bypass the cognitive impairment caused by the disease or a barrier for their independent functioning.^4^

Different professions are researching in this field, adding new levels to the understanding of the needs of elderly people with dementia in their relationship with the surrounding environment. Architects, such as Margaret Calkins, and sociologists, such as John Zeisel, carried out the reviews of the studies in this area and achieved certain principles, or therapeutic objectives,^4^ and design elements such as “exit control, walking paths, common spaces, privacy and personalization, garden access, residential-ness, sensory comprehension, and support for capacity” were correlated with reduced behavioral symptoms.^3^

Nevertheless, the most accessible and least costly ways of adapting to the environment seem to be those made through small interventions. In this context, this review aims to contribute to the development of a better understanding of their impacts, systematizing research evidence.

## METHODS

### Bibliographical survey

This systematic review of the literature was performed in accordance with the preferred reporting items for systematic review and meta-analyses (PRISMA) criteria, and the database searched was MEDLINE/PubMed. In the first place, the combinations between key words for the research were as follows: “*Alzheimer disease OR dementia* AND *wandering* OR *exiting* OR *wayfinding* OR *orientation* OR *room finding* OR *ambulation* OR *mealtimes* OR *dining OR agitation* OR *apathy* AND *interventions* OR *modifications* OR *renovations* OR *environmental* OR *physical environments* OR *door* NOT *review.*” In addition, articles included were peer-reviewed, made in English language scientific literature without data restriction, and are specific to elderly people with dementia and interventions made in *interior* environments.

The exclusion criteria were other systematic reviews, scoping reviews, reviews of literature, animal studies, articles about pharmacological interventions, assessment tools, or concerning interventions not made in the environment, or made in an *external* environment. In addition, the term *dementia* was used to broaden the search, but if the article was explicitly about *only* other types of dementia (e.g., frontal lobe dementia, Parkinson’s dementia, Lewy body dementia, and vascular dementia), it was excluded.

The first author reviewed all keywords, titles, and abstracts of articles from the search results and identified which met the criteria for further review. Both first and second authors reviewed the full articles and reached an agreement in which to include, based on the question “What interventions were made in the interior environment to lessen the behavioral symptoms related to disorientation and to improve social engagement on older persons with probable Alzheimer’s disease?” The third author contributed during the writing process and decision-making.

### Study selection

**Graph 1.**
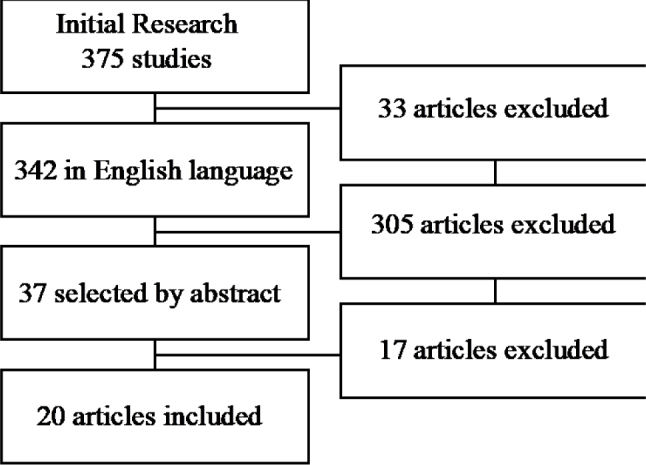
Literature search flow diagram.

The results of the search for the systematic literature review are shown in Graph 1. It is observed that the search strategy resulted in a total of 375 articles, of which only 37 met the criteria for reviewing the full article. Out of these, 20 were qualified. Most of these studies were carried out in the United States (n=5), four studies were carried out in Canada, two in the United Kingdom, two in Germany, three in other European countries, and one in Australia. In addition, three studies did not specify in which country they were developed.

Participants were, primarily, 269 elderly people with dementia, whose type was not usually specified in the articles. However, 50 participants with AD were mentioned, but considering that they are usually 60–70% of the total cases of dementia, we can *estimate* their number to actually reach about 188 elderly people. Beyond them, 57 others participated in the studies, being service providers, as unit managers (n=18), and service users, as family members or nurses (n=39). Three articles did not inform the number of participants involved in their researches, since the studies evaluated the environment itself, were case studies, or considered only three major groups of participants, being families, staff members, and volunteers.

In addition, the search revealed interventions developed in the interior of any care environment, being dementia special care units (SCUs) (n=7), hospitals (n=4), nursing homes (n=5), long-term care facilities (n=2), and adult day care centers (n=2). Interventions comprise modifications in the interior environment that aim to enable the use of the remaining abilities of people with dementia.

## RESULTS

A synthesis table was structured to identify all the selected studies (Table 1), listing the year of the publication, the country in which the study was based, their design, and the journal in which it was published. Another table was created to summarize the results of the review, and the articles were listed with their interventions, objectives, methods, and findings (Table 2). The third table was made to resume and analyze the research evidence collected, highlighting the relations between the type of interventions, their impact on the environment, and their outcomes (Table 3).

**Table 1.** List of articles included following the PRISMA criteria.

Authors and title	Year	Country of study	Study design	Journal
Bautrant et al., Impact of environmental modifications to enhance day-night orientation on behavior of nursing home residents with dementia	2019	France	Brief report	*JAMDA*
Ludden et al., Environmental design for dementia care – towards more meaningful experiences through design	2019	Germany	Case studies	*Maturitas*
Varshawsky et al., Graphic designed bedroom doors to support dementia wandering in residential care homes: Innovative practice	2019	Australia	Pilot project	*Dementia*
Bracken-Scally et al., Assessing the impact of dementia inclusive environmental adjustment in the emergency department	2019	Ireland	Case study	*Dementia*
Hung et al., Do physical environmental changes make a difference? Supporting person-centered care at mealtimes in nursing homes	2017	Canada	Case study	*Dementia*
Wahnschaffe et al., Implementation of dynamic lighting in a nursing home: impact on agitation but not on rest-activity patterns	2017	Germany	Research article	*Current Alzheimer Research*
Hung et al., The effect of dining room physical environmental renovations on person-centered care practice and residents’ dining experiences in long-term care facilities	2016	Canada	Qualitative study	*Journal of Applied Gerontology*
Mazzei et al., Exploring the influence of environment on the spatial behavior of older adults in a purpose-built acute care dementia unit	2014	Canada	Observational case study	*American Journal of AD and other Dementias*
Padilla et al., The effectiveness of control strategies for dementia-driven wandering, preventing escape attempts: a case report	2013	Spain	Case report	*International Psychogeriatrics*
Lancioni et al., Technology-based orientation programs to support indoor travel by persons with moderate Alzheimer’s disease: impact assessment and social validation	2012	Italy	Clinical trial	*Research in Developmental Disabilities*
Barrick et al., Impact of ambient bright light on agitation in dementia	2010	USA	Research article	*International Journal of Geriatric Psychiatry*
Gnaedinger et al., Renovating the built environment for dementia care: lessons learned at the lodge at Broadmead in Victoria, British Columbia	2007	Canada	Case study	*Healthcare Quarterly*
Holmes et al., Keep music live: music and the alleviation of apathy in dementia subjects	2006	United Kingdom	RCT	*International Psychogeriatrics*
Schwarz et al., Effect of design interventions on a dementia care setting	2004	USA	Case study	*American Journal of AD and other Dementias*
Nolan et al., Facilitating resident information seeking regarding meals in a special care unit: an environmental design intervention	2004	-	Clinical trial	*Journal of Gerontological Nursing*
Kincaid et al., The effect of a wall mural on decreasing four types of door-testing behaviors	2003	USA	Clinical trial	*Journal of Applied Gerontology*
Nolan et al., Using external memory aids to increase room finding by older adults with dementia	2001	USA	Clinical trial	*American Journal of AD and other Dementias*
Hewawasam, The use of two-dimensional grid patterns to limit hazardous ambulation in elderly patients with Alzheimer’s disease	1996	-	Case study	*Journal of Research in Nursing*
Dickinson et al., The effects of visual barriers on exiting behavior in a dementia care unit	1995	-	Case study	*The Gerontologist*
Chafetz, Two-dimensional grid is ineffective against demented patients’ exiting through glass doors	1990	USA	Clinical trial	*Psychology and Aging*

**Table 2.** Articles’ main objectives, methods, interventions, and their findings.

Authors	Objectives	Methods	Interventions	Findings
Bautrant et al.^5^	To determine whether environmental rearrangements of the long-term care nursing home can affect disruptive behavioral and psychological symptoms of dementia (BPSD) in residents with dementia	Case study Participants: 19 patients, mean age of 86.3 years. Six patients had Alzheimer’s disease Place: long-term care nursing home Number and duration of disruptive BPSD were systematically collected and analyzed over 24 h or during late hours during each 3-month period	Skylike ceiling tiles in part of the shared premises Progressive decrease of the illuminance at night (6:00–8:00 PM) together with soothing streaming music Reinforcement of the illuminance during the day Walls painted in light beige Oversized clocks in corridors Color (dark blue) of night team clothes different from that of the day team (sky blue)	No significant change in the patients’ dependency, risk of fall, cognitive or depression indexes, or treatment between phases 1 and 2 Number of agitation/physical aggression, screaming, and the mean duration of wandering episodes significantly decreased The number of patients showing wandering was significantly lower, and the mean duration of the episodes decreased, especially during the late hours
Ludden et al.^13^	To show how insights from environmental psychology and advances in technology can inform a user-centered multidisciplinary design approach	Case study Participants: not informed Place: care center for people with dementia A brief meta-review of reviews Two exploratory case studies in which technology-enhanced prototypes were implemented	Six handrails, with different textures, colors, and sounds were designed to match existing scenes along the walking path of the psychogeriatric ward: the sewing room, kitchen, cinema, living room, garden, and farm Installed at the corridor of the ward, and a variety of technology-enhanced nature scenes were designed. All scenes portray a still spacious scene (first layer); a vista to look out over. The second layer comprises a multitude of animated fascinating elements to look at	The textures and colors of the handrails stimulated further exploration and tactile interaction The VR nature scenes were highly successful in promoting a positive and relaxed atmosphere, and in promoting social engagement among residents at the care center and family visiting These designs promoted social engagement (virtual nature), reduced restlessness (both cases), and facilitated wayfinding (experience handrail)
Varshawsky et al.^6^	To observe graphic designed room doors that are visually appealing and to investigate if a design similar to house doors would be a successful approach and environmental change to reduce wandering	Pilot project Participants: Nine residents Place: resident care home Revised Algase Wandering Scale was used to evaluate the effects of the change on wandering (pre- and post-intervention)	Eleven unique custom graphic designs for individual room doors (each door provided multiple approaches to assist with wayfinding and visual recognition: color, location, architectural design, and originality)	Improvement in wandering in the mornings and early evenings Reductions in all behaviors (persistent walking, eloping, and spatial disorientation) were demonstrated after implementation of the new door designs The individuals were observed commenting on the color of their door with visitors to ensure they knew which room they lived
Bracken-Scally et al.^14^	To evaluate the impact of dementia-inclusive modifications made to two emergency department bays in a large acute care hospital	Case study Participants: 10 service users (family carer/member and 16 service providers (staff and key stakeholders) Place: acute care hospital Survey of service providers Interviews with family carers, service providers, and key stakeholders Audit data (at two time points) to evaluate the impact of the modifications	Panels were placed around the walls An electronic display showing the day of the week and time was placed above the entrance Blue and green tones were chosen to replace the clinical white of the walls Standard lighting was replaced with an adjustable system The curtains separating the bays from the corridor were replaced with a movable hard screen Two fixed foldable chairs were installed in each of the bays Storage units were installed Unused equipment was removed	Orientation and navigation within the modified bays were improved though the technical issues with the orientation aid were highlighted This lighting and use of calming colors, together with the addition of noise-reduction bay screens, served to reduce sensory stimulation The provision of adequate space and seating for family carers was extremely beneficial
Chaudhury et al.^15^	To examine the impact of environmental renovations in dining spaces of a long-term care facility on residents’ mealtime experience and staff practice in two care units	Case study Participants: 10 residents at the beginning, 9 at the end, and 17 care aides and nurses Place: A dementia SCU and a non-dementia SCU Pre- and post-renovation ethnographic observations in the dining spaces of the care units and a post-renovation staff survey Four months after the renovations, a staff survey was conducted	Lightning fixtures Wooden-look flooring Decorative items, like vases Wall paintings New height-adjustable tables allowed table height to be altered to accommodate wheelchairs Relocation of the nursing station away from the dining area	In the DEAP, the greatest improvement was the support of the functional ability Proper lighting allowed residents to see their food and tablemates clearly, as well as contributed to a non-institutional and more homelike ambiance Glare continued to be an issue after renovation on both units, which can cause spatial disorientation for residents as they move in and about the space. Regarding the strong color contrast between sections of flooring, it made them think there were stairs and caused them to “avoid walking on the hardwood.” The open kitchen design made the dining area much more obvious to recognize for residents with cognitive impairment The design of having a unit kitchen provided the option of creating a familiar sensory environment related to food and stimulated the residents’ appetite
Wahnschaffe et al.^21^	To test the impact of a dynamic lighting system on agitation and rest-activity cycles in patients with dementia	Research article Participants: 15 residents with dementia Place: nursing home The residents were assessed with the Cohen Mansfield Agitation Index (CMAI) before and after the lighting intervention Rest-activity cycles were monitored for 6 months by using a wrist-worn activity watch	From midwinter, a ceiling-mounted dynamic lighting system was installed in the common room and programmed to produce high illuminance with higher blue light proportions during the day and lower illuminance without blue light in the evening	There were no differences in circadian amplitude and other circadian variables before and after the lighting installation The dynamic lighting in the living room significantly reduced the agitated behavior in demented patients, indicating short-term benefits from higher daily light exposures
Hung et al.^20^	To examine the influences of dining room renovations and enhanced mealtime practices on the quality of residents’ experiences and staff practices	Qualitative study Participants: 12 staff members and 2 unit managers Place: A dementia SCU and a non-dementia SCU Staff focus groups and unit managers’ interviews after the completion of the renovations An assessment tool (DEAP) was developed to conduct a systematic environmental evaluation of the dining rooms in each unit pre- and post-renovations	A dining room with two open kitchens was created Each renovated kitchen was equipped with steam tables and ovens to prepare food The kitchen offered a microwave, fridge, coffee machine, and cabinets of glasses and cutlery. Although the meals were prepared and cooked in a large central kitchen, the unit kitchen had the capacity to cook soup, bake bread and pastries, and so on Furniture and finishing were renewed to enhance homeliness of the dining room New homelike flooring with a wooden look flooring replacing the old vinyl sheet Higher quality recessed lighting and modern ceiling light fixtures were added New dining tables and chairs were brought into the space	Before the renovation, the SCU scored 33 out of 68 in the total score of DEAP. In the post-renovation evaluation with the DEAP tool, the SCU was rated 41/68 The non-SCU scored 29/68 in the total score of DEAP. In the post-renovation, the non-SCU was rated 44/68 in the total score of DEAP Before renovation, noise, lighting, and clutter were major complaints in both dining rooms A supportive physical environment enables people with disabilities greater personal control and autonomy A domestic homelike atmosphere made the place more inviting for social engagement Access to the kitchen, participation in meal-preparing activities, and household chores are not only opportunities for residents to maintain remaining skills but those familiar and meaningful activities can also provide them a sense of achievement, contribution, and inclusion
Mazzei et al.^7^	To examine how the physical environment influenced the spatial behaviors of an understudied population, that is, a small sample of residents living in a traditional acute care hospital, who were then moved to a purpose-built dementia care hospital wing	Case study Participants: Six residents with dementia, ambulatory, and know to engage in aggressive behaviors Place: Two acute care settings from a hospital The data were observational and related to spatial behaviors. In both environments, residents were observed during their most active time of day, mostly between 2 PM and 5 PM	Camouflage murals on exit doorways (depicted as bookcases) Circular wandering path (instead of the previous linear configuration) Private bedrooms with adjoining rooms for the majority of residents (instead of the 4-bed wards) Introduction of an outdoor patio Use of clocks, memory boards, and individual photos in bedrooms or entries to bedrooms, for residents Clutter-free hallway Opportunities for natural light in the unit were increased	Patients spent 24% less time in the nursing station area and more time in their bedrooms and the dining room There is a clear trend toward decreasing numbers of pacing events per day for all residents The wall murals on the door exits had some influence but were not completely effective in masking doors and deterring pacing behaviors. Reasons for this might be that some residents were still cognitively aware of people coming in and out through these doors despite their bookcase camouflage
Padilla et al.^8^	To present effective non-pharmacological intervention strategies for dementia-driven wandering	Case report Participant: an 80-year-old man with AD Place: adult day care center A Spanish translation of the original Algase Scale was used to evaluate wandering behavior	Eight strips of 4 × 105 cm black tape were placed with 4 cm between each other and 25 cm from the exit door Another four strips of the same type were placed on the glass door, 25 cm from the floor	The results showed a significant decrease in wandering behavior frequency in the subject The environmental intervention acted as a subjective barrier to the patient, although the patient was unable to report his subjective perception of the environmental modifications made in the intervention In addition, other residents with dementia with significant cognitive deficits, like our subject, did not approach the area with subjective barriers. It has been shown that every escape attempt was due to a delay of time without receiving attention from the staff
Lancioni et al.^16^	(a) To extend the use of the technology-based program with auditory cues to five new patients with Alzheimer’s disease (b) To compare the effects of this program with those of a program with light cues, to determine whether the latter program could be a viable alternative to the former	Study Participants: Five patients, with lower to moderate AD Place: day center Within each session, a patient was to reach five of those destinations/rooms to deliver and/or pick up small objects and meet a staff person present there The measures recorded during the travel sessions were (a) the travels programmed and whether they were carried out correctly and (b) the duration of the travels	A system with auditory cues included a sound source at each of the destinations and a portable control device to activate and deactivate those sources. The recordings available consisted of short sentences encouraging the patient to walk to the destination A system with light cues differed in that light sources replaced the sound sources. Each light source contained two green strobe lights, which emitted approximately one flash per second until the patient reached the destination	Both program conditions were effective from the initial sessions. The mean percentages of correct travels varied between slightly below 90 and over 95 Psychology students provided higher scores for the program using light cues on all six items of the questionnaire
Barrick et al.^23^	To evaluate the effect of ambient bright light therapy on agitation among institutionalized persons with dementia	Research article Participants: 66 older persons with dementia Place: a psychiatric hospital unit and a dementia-specific residential care facility Outcome measures included direct observation by research personnel and completion by staff caregivers of the 14-item, short form of the Cohen-Mansfield Agitation Inventory (CMAI)	High intensity and low glare ambient lighting was installed in activity and dining areas	Analyses of observational data revealed that for participants with mild/moderate dementia, agitation was higher under AM light, PM light, and all daylight than standard light There was a trend toward severely demented participants being more agitated during AM light than standard light In no comparison was agitation significantly lower under any therapeutic condition, in comparison to standard lighting
Gnaedinger et al.^22^	To improve the quality of care and of life for veterans with dementia by renovating the existing dementia care lodges in ways that reflect a new awareness of the impact of the built environment on persons with dementia.	Case study Participants: not informed Place: a lodge inside a geriatric residential care facility Staff members, families, and volunteers were surveyed for their observations and opinions after renovations were complete	The 32-bed was separated into two smaller lodges A new homelike kitchen, living room, and dining room were built Painting murals were used to camouflage exit doors in common areas Non-institutional finishes and furnishings were used A silent resident call system was installed	All three groups surveyed remarked that the lodges are now more homelike, pleasant, calm, quiet, relaxing, and welcoming Residents are “really more at peace” and are engaging in more “normal” behavior, such as curling up on a couch by a fireplace or participating in making tea with a family member in the kitchen Ratings of residents’ quality of life increased Design team members should give consideration to lighting as a means of attracting residents to preferred living areas
Holmes et al.^19^	To explore whether music, live, or prerecorded is effective in the treatment of apathy in subjects with moderate to severe dementia	RCT Participants: 32 subjects with moderate to severe dementia and with diagnostic criteria for apathy Place: subjects were recruited from residential and nursing homes Each subject was randomized to 30-minute music or silent periods and was video recorded, and the muted recording was analyzed every 3 min using dementia care mapping to assess the quality of engagement to the blinded music intervention	The communal area of the residential-care or nursing-home facility was used for the music intervention. Music periods comprised three different activities, each of 30 min duration. One 30-min period consisted of silence alone, one 30-min period consisted of the playing of background prerecorded music, and one 30-min period consisted of the playing of live music from session musicians	The majority of subjects (69%), regardless of dementia severity, showed a significant and positive engagement to live music Engagement to prerecorded music was nonsignificant, with just 25% of all subjects showing positive engagement No subjects showed any evidence of experiencing a state of ill-being during either the live or prerecorded music sessions
Schwarz et al.^24^	To determine whether design interventions affect desirable behavioral outcomes in nursing home residents with dementia	Case study Participants: not informed Place: long-term care facilities Pretest and post-test design for data collection and a combination of quantitative and qualitative methods PEAP was used to conduct the focused evaluation of the facility before and after environmental modifications Two focus group interviews were conducted with facility staff members	The architecturally dominant central nurses’ station was replaced with an aviary, introducing a smaller nurses’ station The dining areas were decentralized for smaller groups of residents The interior design was improved by adding appropriate lighting and carpeting	The newly built cluster arrangement scored higher in all eight areas of the PEAP instrument compared with the scores of the facility before renovation The three areas of (1) maximize awareness and orientation, (2) provision of privacy, and (3) facilitation of social contact had the highest variation in the pre- and post-renovation PEAP scores The general reaction from the staff was that replacing the prominent nurses’ station with the aviary reduced the institutional ambiance in the facility However, some staff members were concerned that residents sitting near the aviary were engaged in passive behavior
Nolan et al.^18^	To evaluate the effect of an environmental modification designed to provide residents of a special care unit with easy access to information about mealtimes	Clinical trial Participants: 35 residents with AD Place: SCU at a nursing home An ABAB reversal design across mealtimes was used to determine whether the intervention changed the frequency of residents’ requests for food or meal-related statements before mealtimes	Large clock (d=16 in) hung in the dining room Large-print sign (22 × 28 in) that identified mealtimes in the dining room hung below the clock, constructed of poster-board	Similar effects of the treatment were replicated across all three mealtimes (breakfast, lunch, and dinner) The intervention decreased residents’ repetitive statements and questions regarding food and mealtimes The staff stated that they believed the signs that helped them reduce residents’ pre mealtime confusion and agitation
Kincaid et al.^11^	To examine the effect that a wall mural painted over an exit door had on decreasing door-testing behaviors of residents with dementia	Study Participants: 12 residents with a diagnosis of dementia Place: SCU at a nursing home Data were collected both before and after the wall mural was painted The door-testing behavior was the dependent variable, and the physical appearance of the entrance/exit doorway was the independent variable	Wall mural painted on the entrance/exit doorway to disguise it. It is a two-door with windows that need to remain functional, only opened by a keypad. It was painted from the floor to the ceiling, covering the doors and adjoining walls	The findings indicate that when a wall mural is painted over the entrance/exit doorway, the frequency of door testing does decrease Out of the 12 residents who were active at the doors, only 3 remained active at the doors after installation of the wall mural Two types of door-testing behaviors decreased significantly after the installation of the wall mural. Type 1: walking up to the door and pushing or pulling calmly and Type 2: using a team effort, which had a significant decrease
Nolan et al.^17^	To evaluate the impact of placing two external memory aids outside participants’ bedrooms	Multiple-baseline experiment Participants: Three residents with AD Place: SCU at a nursing home for people with dementia Each resident’s ability to locate her own room was assessed by using a direct observation technique A multiple-baseline design across subjects was used to evaluate the effect of the photograph and sign on room finding	A portrait-type photograph from early adulthood and a large-print sign with a sentence indicating the resident’s name were both placed outside each study participant’s room	All participants improved during the intervention phase. There was over a 50% mean increase in participants’ ability to accurately locate their own room following the intervention
Hewawasam^12^	To capitalize on the observation that many individuals who suffer from dementia of Alzheimer’s type appear to perceive two-dimensional patterns as barriers	Study Participants: 10 patients with mean to severe dementia Place: NHS trust hospital ward for the elderly mentally infirm The design was based on an ABABA single-subject design that incorporated several baseline (control) observations, one before and one after each experimental manipulation	Of note, 3.8 cm strips of black tape applied 3.8 cm apart to the blue vinyl floor, extending in front of the exit door. They were applied in one of two configurations, grid A – horizontally, and grid B – vertically	All 10 patients showed varying degrees of changes to their normal gait while crossing the grid. These changes were manifested by some hesitation and deliberation before crossing and/or stepping over the eight-strip Five patients, of which four had a diagnosis of AD, showed a statistically significant reduction in the number of door contacts
Dickinson et al.^9^		Study Participants: Seven residents diagnosed with AD or other types of dementia Place: dementia care unit	Horizontal mini-blind on the window panels in the exit doors, blue (surrounding door/door frame) Cloth barriers, with cotton fabric, also blue	Exiting decreased 44% with the blind closed With just the cloth barrier, exiting decreased dramatically, for a reduction of 96% With both the blind and cloth barrier, attempted exits decreased 88%
Chafetz^10^	To extend the findings of Hussian and Brown (1987) to a nursing home setting	Research study Participants: 30 residents, all with some type of dementia Place: long-term care unit ABA research design Continuous frequency data were collected by unit staff. The dependent variable of main interest was the frequency of door openings, as indicated by the sounding of the buzzer when a resident opened either exit doors	Placement of eight strips of black plastic tape on the floor, parallel with the door threshold	The clear ineffectiveness of the grid in this setting confirms that individuals with dementia will cross the grid on their way to a glass door or a double-wide door Glass doors allow residents a full view of the visually attractive and physically unrestricted spaces that lie beyond, therefore distracting the residents’ attention from the grid

**Table 3.** Overview of environmental characteristics, related interventions, and the respective outcomes.

Environment		Wandering	Exiting	Door testing	Wayfinding	Social engagement	Agitation	Functional ability
Communications	Camouflage	ø	-	-				
	Signage	-			+		- - - -	
	Cues	-	-		+ + +			
	Barriers	-	ø	-				
Features	Light	-			+	+ +	ø -	
	Music	-				+		
	Furniture					+		+
	Reduced sensory stimulation				+ +			
	Homelike finishes and fixtures	-			+	+		+ + +
	Virtual					+		

The number of studies is indicated by the number of symbols in each field; “+” indicates the increase of the outcome and “-“ indicates the decrease of the outcome. The “ø” indicates an absence of impact in the outcome.

References to home furnishings and finishes, which were used to improve functional capacity, social engagement, and wayfinding, were widely cited in the researched articles. These findings are in agreement with Zeisel, who suggested homemade qualities (i.e., decoration, furniture, and lighting) to reduce aggression and other symptoms.^3^ Cues are usually utilized to suggest appropriate spatial behaviors,^3^ and this type of intervention had compatible results from this review: the major impact was on the wayfinding of the residents, reducing behaviors of exiting and wandering. The use of signage also proved to be an effective intervention, reducing agitation and wandering and improving wayfinding.

## DISCUSSION

Patients who are unable to identify the paths to the desired locations experience anxiety, confusion, mutism, and even panic.^4^ In contrast, even people with dementia walk with purpose when they are able to understand where they are and where they are going.^3^ Thus, among the studies listed in this review, 12 aimed to intervene in this theme in some of its dimensions. There are interventions to reduce ambulation and excessive stimulation of the patient,^5^ ^‒^ ^8^ attempts to prevent exit or escape,^6^ ^,^ ^8^ ^‒^ ^10^ door testing,^11^ ^,^ ^12^ and improvements in orientation/location.^6^ ^,^ ^7^ ^,^ ^13^ ^–^ ^17^

But how do people with dementia know where to go? The answer is when they manage to perceive the next object of place. Like everyone else, they move around unknown places through landmarks. Therefore, they need a place that communicates with them. In this sense, the interventions that had this purpose were classified as environmental communications. Among them were *camouflages* (n=3), *tracks* (n=9), *barriers* (n=3), and *signs* (n=3).

Camouflage interventions are those that try to hide the exits, whether portraying them as shelves,^7^ mini-blinds or panels,^9^ or as a painted mural.^11^ The last two had positive impacts, as they reduced the frequency of exit attempts and door testing. The first was not entirely effective in stopping such behaviors, and one of the reasons attributed to this fact was that some residents were still cognitively aware of the entry and exit of people through the doors, despite a camouflage from the bookcase. These strategies are in line with the findings of Zeisel^4^ who stated that some doors attract the natural curiosity of the human brain and should be less inviting, as invisible as possible.

The signaling interventions identified were the landmarks placed in the environment. Among them were six handrails, with different textures, colors, and sounds, designed to match the neighboring rooms (i.e., kitchen, cinema, sewing room, living room, garden, and farm).^13^ These handrails facilitated orientation and reduced restlessness.

Contrary to the previous proposal to hide doors to unsafe places, doors to safe destinations should be as inviting as possible.^3^ One of the interventions proposed bedroom doors with a personalized design, with positive results both in ambulation and in behaviors (reducing output and improving wayfinding).^6^ Two other types of interventions using light and hearing aids were tested, and both conditions were effective in improving the wayfinding. However, the results of the light cues scored higher.

In the field of signs, the use of clocks^5^ ^,^ ^7^ ^,^ ^18^ was introduced to guide residents in time. In addition, a portrait photo of early adulthood and a large-letter sign with a phrase indicating the resident’s name were placed outside the room of the participants in one of the studies.^17^ In particular, personal items that refer to the past, achievements, and social roles help people with dementia to support their sense of identity.^3^ With this in mind, this study demonstrated improvements, increasing the participants’ ability to locate their rooms by more than 50%.

Barrier interventions focused on creating obstacles to prevent people with dementia from trying to access places they should not have to. Thus, taking advantage of the fact that people with AD have a deficiency in contrast sensitivity,^4^ three studies used strips of black tape, with different measures and distances, on the near floor and on the exit doors.^8^ ^,^ ^10^ ^,^ ^12^ Two studies had positive results, showing a significant decrease in ambulation^8^ and in the number of door contacts.^12^

Contrary to these, another study^10^ showed no effects. This negative result was attributed to the fact that the intervention was carried out on a glass door that allowed residents “a complete view of the visually attractive and physically unrestricted spaces that are beyond,” thus distracting them from the grid. In addition, in another article,^15^ it was mentioned that individuals with dementia avoided walking on the wooden floor. This was interpreted as a reaction to the strong contrast of colors created between the sections of the floor, which made them think they were stairs.

Still, on the topic of contrast deficiency in people with dementia, an intervention sought, by means of panels placed around the walls, to add enough contrast to help them distinguish breaks between walls and floors and between objects and their background.^14^ This intervention had positive results, increasing its capacity for orientation. In the same study, the walls were painted in shades of blue and green to replace an earlier clinical white color. In this case, the shades of blue and green are considered calming colors. The observed result was a reduction in sensory stimulation. Another study painted the walls in light beige,^5^ which along with other modifications, highlighted the daytime and nighttime orientation of residents.

Color selection also plays a role in the movement toward deinstitutionalization. The term non-institutional was widely used among the reviewed articles. Calkins^3^ warned that the use of the preposition does not designate what design should or should not be. Despite this, some designers use the term homemade, which assumes elements such as wood instead of metal or plastic and a style that would be used in someone’s home (although there is no such style).

Interventions related to the characteristics and atmosphere of the space were classified as environmental characteristics. Within these, some articles addressed the issues of light (n=6), music (n=1), home finishes and accessories (n=5), sensory stimulation (n=3), furniture (n=6), and virtual environment (n=1). Its results, in addition to improving the wayfinding,^5^ ^,^ ^7^ ^,^ ^14^ ^,^ ^15^ were also in the social involvement of the person with dementia,^7^ ^,^ ^13^ ^,^ ^19^ functional capacity,^15^ ^,^ ^20^ ^,^ ^21^ and agitation behaviors.^22^ ^,^ ^23^

Among the reviewed articles, this was exactly the point: mural paintings and wooden floors were, in fact, some of the proposed interventions.^5^ ^,^ ^14^ ^,^ ^15^ ^,^ ^20^ ^,^ ^21^ These modifications are located on the topic of finishes and home accessories. The replacement of the floor by a wood-type floor was made in dining rooms,^15^ ^,^ ^20^ ^,^ ^21^ kitchens, and living rooms^21^ to complement the family environment that resulted in increased social engagement.

There was also an article^24^ in which the nurses’ central post was replaced by an aviary, and adjustments were made to “make it less institutional,” such as the use of rugs. The dining and kitchen areas, in this case, were decentralized and divided into three smaller ones, for 10–12 residents, which proved to be able to enhance social contact and guidance.

This family atmosphere was complemented by the implementation of new furniture, with fully renovated kitchens and the ability to prepare quick meals, such as making soup or baking bread.^15^ ^,^ ^20^ ^,^ ^21^ In one of the articles,^21^ it was emphasized that “residents were more at peace,” and engaged in behaviors such as participating in tea making with a family member. Two others demonstrated that an open kitchen can be more obviously recognized, creating a familiar sensory environment related to food and stimulating the residents’ appetite.^15^ ^,^ ^20^

In addition to the search for a family environment, the installation of new tables that allowed changing the height to accommodate wheelchairs proved to have a great influence on the functional support capacity.^15^ In contrast, the installation of fixed chairs in the stalls of an acute care hospital^14^ had a positive impact on social engagement, allowing family caregivers to be with the elderly people for longer and more comfort.

On the issue of social engagement, scenes of virtual nature were projected in the corridor of an infirmary at a service center.^13^ This intervention created a relaxed atmosphere that stimulated social involvement not only among residents but also with visiting family members and, additionally, reduced agitation behaviors.

Dementia along with old age weakens the signals sent to the brain by each sense individually.^4^ This makes it more difficult for elderly people to understand the environment around them. Two studies addressed this issue, installing storage units^14^ and a so-called silent resident system,^21^ removing unused equipment, and replacing the curtains that separated the bays of an intensive care hospital with rigid mobile screens.^14^ These interventions alleviated the confusion and helped patients to better understand their environment.

Regarding the light theme, two studies focused on the impact of bright light on night sleep and daytime involvement. In one of the interventions researched in this review,^23^ high-intensity and low-brightness ambient lighting was installed in common areas, such as the activity room and the dining room. The analysis of the collected data demonstrated that the agitation was not significantly less in the therapeutic condition, in comparison with the standard lighting.

Another study installed a ceiling-mounted dynamic lighting system in a common area, programmed to produce high light during the day and low light at night. Although it did not impact amplitude and other circadian variables, dynamic lighting significantly reduced agitation in patients with dementia. The standard lighting replaced by an adjustable system^14^ served as an element in improving the wayfinding and reduced unnecessary sensory stimulation. The improved lighting^15^ ^,^ ^24^ also allowed residents to see their food and their tablemates clearly, contributing to social engagement and food intake.

One study increased the possibilities of natural light^7^ and another reinforced the light during the day and progressively decreased the light at night, together with the streaming of soft music.^5^ The results showed that the number of episodes of agitation and the average duration of episodes of wandering decreased significantly. In addition to this intervention with music, another study indicated that, regardless of the severity of dementia, exposure to live music is related to positive engagement.^19^ This study also emphasized that an exposure to prerecorded music has no significant effects.

**Graph 2.**
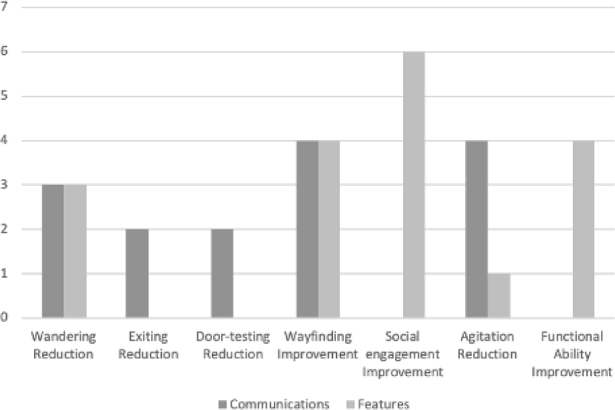
Number of interventions with successful results related with their outcomes.

As shown, environmental communication seems to be used to influence orientation behaviors, being the only type of intervention aimed at the exit and door testing, also bringing positive results (Graph 2). They were also used to impact the reduction of wandering and the improvement of the wayfinding, and the results of which environment resources were also used. However, in the search to reduce agitation, environmental communication seemed to be more used. The environmental characteristics, such as the home climate brought by finishes, utensils, and furniture, were more applied in the search for improvements in social engagement and functional capacity (Graph 2).

The evidence collected illustrates the relevant impact of environmental interventions on the behavior of elderly people with AD. Most of the researched studies showed that to impact the orientation of the elderly people, the environment must communicate with them, but to influence their social behavior, the characteristics of the environment must be updated, usually bringing a more homely aspect.

In any case, the use of elements from the two major intervention groups can improve the overall quality of life of patients with dementia. However, it should be noted that the changes in the physical environment must be monitored by the team, not only using it as a source of information but also training them to know how to support the person in this new environment.

Overall, this review showed a variety of possibilities for improving the interaction of people with dementia with the environment in which they live. Capacity-building strategies in the physical environment allow them to naturally use their remaining skills, remaining independent for a longer time, and therefore improving their senses of themselves. The limitations of this research mainly include the fact that most studies use a multimodal approach, making it difficult to determine the specific impact of which intervention.
